# Estimating the volume of dirty money in Iran

**DOI:** 10.1016/j.mex.2020.101074

**Published:** 2020-09-28

**Authors:** Anita Dowlatzadeh, Hossein Akbarifard

**Affiliations:** Department of Economics, Faculty of Management and Economics, Shahid Bahonar University of Kerman, Kerman, Iran

**Keywords:** Dirty money, Fuzzy logic, VAR model, VECM model

## Abstract

In this study, the volume of dirty money in Iran was estimated. The data belonged to the period of 1997-2019, and was taken from the Central Bank of Iran (website: https://www.cbi.ir). Fuzzy logic was used to estimate the underground economy. Fuzzy theory can mathematically formulate many variables that are imprecise and ambiguous concepts. This theory is appropriate for reasoning, inference, control, and decision-making under uncertainty. This approach works in conditions of uncertainty. In cases in which the variables are inaccurate, this method is used. Fuzzy set theory is a generalization of the set theory. The underground economy is important in estimating the amount of dirty money and has a positive effect on this amount. The effect of the underground economy was investigated using the vector autoregressive (VAR) and vector error correction (VECM) models.

• In this article applied the fuzzy logic, to estimate the underground economy.
• The method presented in this article can be useful for Researchers and managers in the monetary trend of economics.
• The fuzzy method is the best way to estimate the size of the underground economy because it is a measure of uncertainty.

## Graphical abstract

**Figure:**
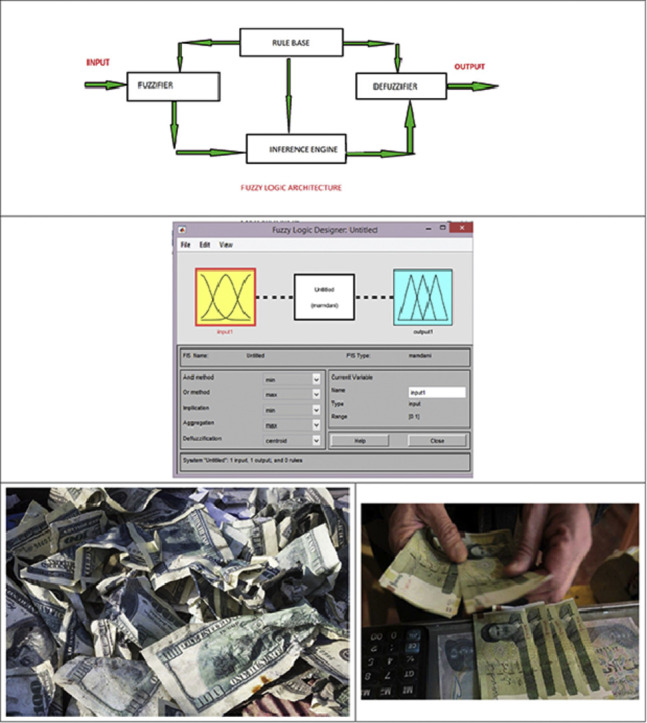

Specifications table

**Table:** 

Subject Area:	Select one of the following subject areas:• Economic science
More specific subject area:	Informal economy; Underground economy
Method name:	Estimating the volume of dirty money/ Fuzzy logic
Name and reference of original method:	Fuzzy theory can mathematically formulate many variables that are imprecise and ambiguous concepts. Zadeh, L. A. (1965). “Fuzzy sets”. Information and control 8, pp 338- 353.
Resource availability:	If applicable, include links to resources necessary to reproduce the method (e.g., data, software, hardware, and reagent)

## Method details

The study of money demand behavior in a specific economy has immense importance in the macroeconomic context [1]. Mt is the total demand for money at the time of *t* [2]. The interest rate and the informal market exchange rate were used as the exchange rate variables in the model [3]. The volume of liquidity was obtained by dividing the amount of money by the retail price index, which is always nonnegative. Gross domestic product (GDP) was considered at the fixed price of the year 2005.

**Fig. 1:**
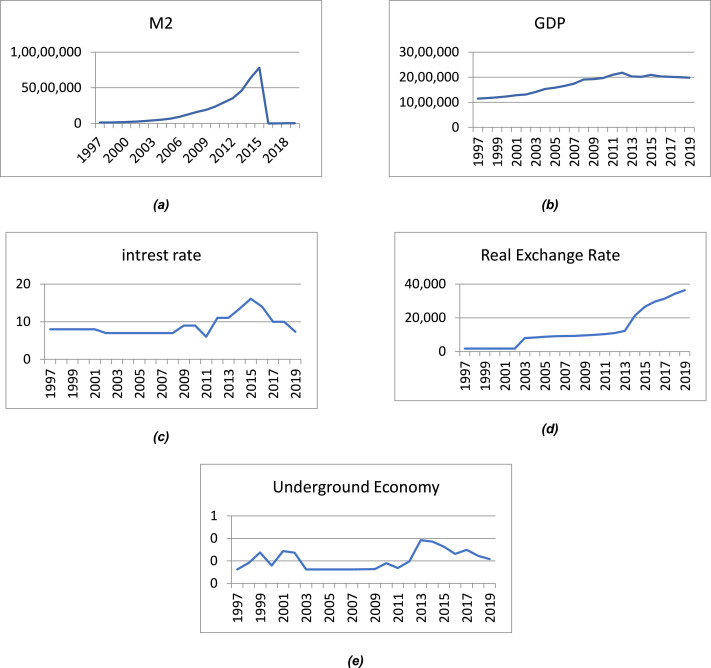
Input data to the fuzzy model: (a) M2, (b) GDP, (c) Interest rate, (d) Real exchange rate and (e) underground economy.

**Fig. 2:**
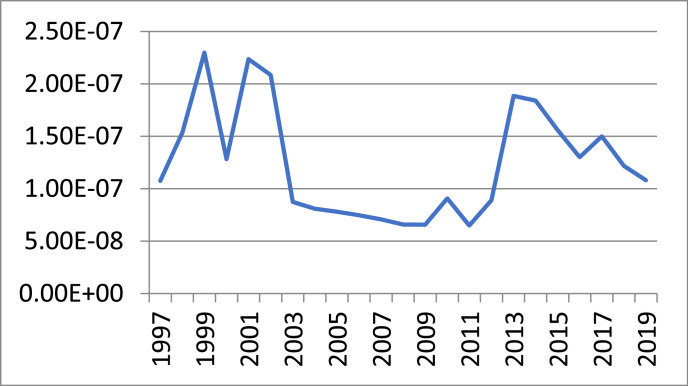
The graph of underground economy.

**Fig. 3:**
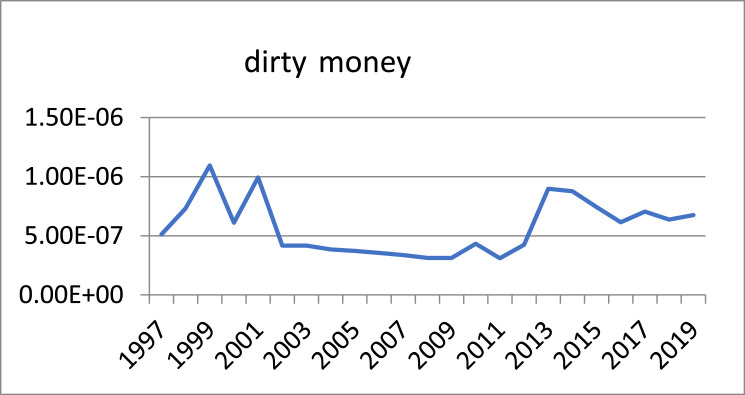
The Graph of the volume of dirty money.

The nature of the underground economy is hidden, since activities in the underground economy are not reported. Therefore, as previously mentioned, direct methods cannot be used to estimate the size of an underground economy. In this paper, a fuzzy approach is proposed to obtain this estimate. Indirect methods should be used to estimate the size of the underground economy. The fuzzy method is one of the approaches used to work in conditions of uncertainty. It is a form of multi-valued region in which the logical value of the variables can be any real number between 0 and 1 as well as these numbers themselves. The underground economy is estimated using the fuzzy method. In this study, in order to estimate the volume of dirty money, the data from 1997 to 2019 were taken from the Bank of Iran (website: https://www.cbi.ir). (Fig. 1a) indicates the graph of the variable forming the total demand for the money. Fig. (1b) indicates the graph of the variable forming the GDP. Fig. (1c) shows the graph of the variable forming the interest rate. Fig. (1d) shows the graph of the variable forming the real exchange rate. Fig. (1e) shows the graph of the variable forming the underground economy (Figs. 2 and 3).

## Experimental design, materials, and method

In this section, the underground economy is estimated first. Professor Zadeh in his paper “Fuzzy sets” introduced the concept of the first fuzzy logic in 1965. This was the first step to the fuzzy approach. The fuzzy theory can mathematically formulate many variables that are imprecise and ambiguous concepts. This theory is appropriate for reasoning, inference, control, and decision-making under uncertainty. This approach can work in conditions of uncertainty. In cases in which variables are inaccurate, this method is used. Fuzzy set theory is a generalization of the set theory. In the conventional set theory, elements either belong to the set or not. If the membership exact element is not specified, the fuzzy method can be used. This method assigns a number between [0, 1] to each element and that number is the membership degree. For example, assuming that X is a reference collection, A subsidiary is defined as follows:

(1) A={x∈X|P(x)}

In Eq. (1), *P(*x) has a well-defined property, but P(*x*) cannot be well-defined. For example, *X* is the set of natural numbers and *P* is the characteristic of much less than 15. These cases are difficult to model. The variable is assumed to be accurate. If *A*⊂*X* and *x*ϵ*A*, then we have:

(2) $$\mu_{A}\left(x\right)=\left\{ \begin{array}{cc} 1, & x\in A\\ 0, & x\notin A \end{array}\right.$$

Fuzzy set *A* in *X* is characterized by the membership function *f_A_*(*x*), which associates with each point in *X*, a real number in the interval [0,1] with the value of *f_A_*(*x*), with x representing the “grade of membership” of *x* in *A*. Thus, the nearer the value of *f_A_*(*x*) is to unity, the higher the grade of membership of *x* is in *A*. When *A* is a set in the ordinary sense of the term, its membership function can take on only two values of 0 and 1; *f_A_*(*x*) = 1 or 0, according to *x*, does or does not belong to *A*. Thus, in this case, *f_A_*(*x*) reduces to the familiar characteristic function of set *A* [4], according to Sol et al. [7].

The interrelationship between the `hidden economy' and money demand is derived from the maintained hypothesis consisting of the following:

(i) The unrecorded economy almost entirely uses money for transactions.
(ii) The money demand has two distinct components, namely, the demand from (a) the recorded economy and (b) the unrecorded economy.
(iii) The set of variables included to explain the variations in the money demand of the recorded economy is complete and exhaustive.
(iv) There is no functional misspecification in the recorded sector demand for the money.

Thus, following the maintained hypothesis, the following equation can be written:

(3) $$M_{t}=M_{Rt}+M_{URt}$$

where *M_t_* is the total demand for money at the time of *t*, and *M_Rt_* and *M_URt_* are the demands from the recorded and the unrecorded economy, respectively. The income generated in the unrecorded economy is termed the `hidden economy' in this paper (see Footnote 2). A detailed discussion of the issues related to this concept is available in the studies by Feige and Tanzi [5,6]. The money demand equation for the recorded sector is specified following the `optimum cash balance' theory. For a recent discussion of this theory see Baumol and Tobin. The specification used in this study is a generalized form of the model that follows the `optimum cash balance' theory with an extension to incorporate the dynamic structure (or out of optimum holdings) of the individual behavior. Thus, the money demand equation for the recorded sector is:

(4) $$M_{Rt}=\alpha_{1}Y_{Rt}^{\beta_{1}}R_{t}^{\beta_{2}}P_{t}^{\beta 3}e^{F\left(L\right)u_{t}},$$

where *M_Rt_* is as defined before, *Y_Rt_* is the recorded income variable, *Rt* is the short-term interest rate, and *Pt* is the retail price index. Moreover, a, IlNl A2, and fl are the parameters and *F*(*L*) is a polynomial in the lag operator *L*. It is also assumed that:

(5) $$E\left(w_{t}\right)=0,E\left(w_{t}w_{s}\right)=\sigma_{w}^{2}\;for\;t=s\;and\;zero\;otherwise.$$

It is also assumed that *E*(ut ws) = o for all the t and s. The parameter β4 is unknown but expected to be near unity.

The variables MRt, MURt, and Yht are individually unobservable; however, MRt+MURt is observable. Hence, by combining Eqs. (4) and (5) and then taking logarithms and approximating by using the linear terms of a Taylor expansion, the model becomes:

(6) $$m_{t}=ln\alpha_{1}+\beta_{1}y_{Rt}+\beta_{2}r_{t}+\beta_{3}p_{t}+F\left(L\right)u_{t}+\left(Y_{ht}^{\beta_{4}}+w_{t}\right)/f\left(.\right),$$

where

$$f\left(.\right)=\alpha_{1}Y_{Rt}^{\beta_{1}}R_{t}^{\beta_{2}}P_{t}^{\beta_{3}}e^{F\left(L\right)u_{t}}$$

and logarithms of all capital letters are denoted by small letters. Denoting F(L) ut by єt, the disturbance terms of Eq. (6) can be written as:

(7) $$\in_{t}+w_{t}/f\left(.\right).$$

Due to the assumption of the independence of ut and wt, the disturbances єt and wt are also independent of each other. It can be shown that the disturbance structure in (7) is approximately:

(8) $$\in_{t}+v_{t}$$

where *E*(є*t*) = *E*(*vt*) = o; and *E*(*vtvs*) = *σ*^2^_v_ for *t* = *s* and zero otherwise; *E*(є*t, vs*) = o for all the *t* and *s*. By definition, єt has an autoregressive structure, the order of which depends on *F*(*L*). It is assumed that *E*(є*t* 2) = *σ*^2^_є_, hence Eq. (8) can be rewritten as:

(9) $$m_{t}=ln\alpha_{1}+\beta_{1}y_{Rt}+\beta_{2}r_{t}+\beta_{3}p_{t}+F\left(L\right)u_{t}+\left(Y_{ht}^{\beta_{4}}\right)/f\left(.\right)+\in_{t}+v_{t}.$$

It is easy to see that Eq. (9) is a hybrid specification and markedly different from the standard specifications used in the literature for the money demand. In particular, the authors noticed that it is not a constant-elasticity model and that the short-run elasticities of money demand with respect to interest rate, price level, and total income (recorded + hidden) are as follows:

Interest elasticity is:

(10a) $$\mu_{mr}=\beta_{2}\left(I.o-Y_{ht}^{\beta_{4}}\right)/H\left(.\right).$$

Price elasticity is:

(10b) $$\mu_{mp}=\beta_{3}\left(I.o-Y_{ht}^{\beta_{4}}\right)/H\left(.\right).$$

Income elasticity is:

(10c) $$\mu_{my}=\beta_{1}\left(\partial y_{Rt}/\partial y_{t}\right)+\left[Y_{ht}/H\left(.\right)\right]\left[\beta_{4}\left(\partial y_{ht}/\partial y_{t}\right)+\beta_{1}\left(\partial y_{Rt}/\partial y_{t}\right)\right]$$

where

(11) $$H\left(.\right)=\alpha_{1}Y_{Rt}^{\beta_{1}}R_{t}^{\beta_{2}}P_{t}^{\beta_{3}}$$

It is also important to note that, for our model

(12) $$\left(\partial m_{Rt}/\partial p_{t}\right)>0\;\text{does}\;\text{not}\;\text{imply}\;\text{that}\left(\partial m_{t}/\partial p_{t}\right)>0,$$

and

(13) $$\left(\partial m_{Rt}/\partial r_{t}\right)<0\;\text{does}\;\text{not}\;\text{imply}\;\text{that}\left(\partial m_{t}/\partial p_{t}\right)<0.$$

Hence, the point estimates for the parameters of the model may look as if they had conflicting signs in the conventional sense [1].

(14) $$LM_{t}=a+bLy_{t}+cR_{t}+dLEXR_{t}+eLyu_{t}+\varepsilon_{t}$$

## Estimating the underground economy of the fuzzy model

If the variable is accurate, it can be any value attributable to a membership degree. After determining the membership functions, the rules are made. Decision rules are made based on the criterion of “if-then”. The number of decision rules depends on the number of input variables and the fuzzy membership sets. For example, if the number of input variables is 2 and the basic elements of a fuzzy set are 5 and 25, the rule is thus made. We have 25 rules (Table 1, Table 2, Table 3, Table 4).

**Table 1:** Fuzzy rules.

Rules	TR	NR	Underground economy	Weight
1	VH	VH	VB	1
2	VH	H	VB	0.8
3	VH	N	S	1
4	VH	L	S	0.8
5	VH	VL	A	0.8
6	H	VH	VB	1
7	H	H	B	1
8	H	N	B	0.8
9	H	L	A	1
10	H	VL	S	1
11	N	VH	B	1
12	N	H	B	0.8
13	N	N	A	1
14	N	L	S	0.8
15	N	VL	S	1
16	L	VH	B	1
17	L	H	A	1
18	L	N	S	0.8
19	L	L	S	1
20	L	VL	VS	1
21	VL	VH	A	0.8
22	VL	H	S	0.8
23	VL	N	S	1
24	VL	L	VS	0.8
25	VL	VL	VS	1

VH: Very high, H: High, N: Normal, L: Low, VL: Very low.

VB: Very big, B: Big, A: Among, S: Small, VS: Very small.

The table of fuzzy rules is written according to the article by Hui Kuang Yu et al. (2006) [8].

**Table 2:** Input and output variables.

Year	Tax burden rate (TR)	Exchange rate margin	Underground economy/GDP
1997	0.046338881	2692	1.07473E-07
1998	0.054935087	3027	1.53599E-07
1999	0.0704991	4713	2.29732E-07
2000	0.08517605	6879	1.2821E-07
2001	0.058621673	6376	2.23529E-07
2002	0.057147996	6170	2.08468E-07
2003	0.049879606	33	8.74872E-08
2004	0.052331648	41	8.0865E-08
2005	0.053803409	28	7.8082E-08
2006	0.067501023	19	7.46643E-08
2007	0.062966648	31	7.07235E-08
2008	0.061095341	72	6.58842E-08
2009	0.064044821	93	6.5761E-08
2010	0.077056679	59	9.06982E-08
2011	0.060009434	262	6.49408E-08
2012	0.05755123	2606	8.90715E-08
2013	0.055724867	13799	1.88562E-07
2014	0.052900117	10586	1.84069E-07
2015	0.06566305	6292	1.55862E-07
2016	0.061882238	4921	1.30117E-07
2017	0.069240491	5051	1.49989E-07
2018	0.073731552	2218.5	1.21693E-07
2019	0.078255583	420.9	1.08019E-07

**Table 3:** The VAR model.

Year	LM2	b	LY	c	LR	d	LEXR	e	Lyu
1997	10.15759	4.080285	13.95394	-1.806800	2.079442	1.524667	8.39976	0.591130	-2.09209
1998	10.1404	4.080285	13.97412	-1.806800	2.079442	1.524667	8.472614	0.591130	-1.7148
1999	10.15157	4.080285	13.99319	-1.806800	2.079442	1.524667	8.774622	0.591130	-1.29316
2000	10.1518	4.080285	14.0244	-1.806800	2.079442	1.524667	9.063463	0.591130	-1.8452
2001	10.28995	4.080285	14.06445	-1.806800	2.079442	1.524667	9.003439	0.591130	-1.24927
2002	10.43549	4.080285	14.08435	-1.806800	1.94591	1.524667	8.977778	0.591130	-1.29913
2003	10.55196	4.080285	14.16128	-1.806800	1.94591	1.524667	8.986071	0.591130	-2.09049
2004	10.63859	4.080285	14.24199	-1.806800	1.94591	1.524667	9.026778	0.591130	-2.08849
2005	10.76126	4.080285	14.27501	-1.806800	1.94591	1.524667	9.076466	0.591130	-2.09049
2006	10.95765	4.080285	14.31977	-1.806800	1.94591	1.524667	9.109636	0.591130	-2.09049
2007	11.17778	4.080285	14.374	-1.806800	1.94591	1.524667	9.129781	0.591130	-2.09049
2008	11.25332	4.080285	14.46114	-1.806800	1.94591	1.524667	9.14388	0.591130	-2.07422
2009	11.17492	4.080285	14.47085	-1.806800	2.197225	1.524667	9.176473	0.591130	-2.06638
2010	11.28712	4.080285	14.49321	-1.806800	2.197225	1.524667	9.208238	0.591130	-1.72252
2011	11.39475	4.080285	14.55606	-1.806800	1.791759	1.524667	9.268704	0.591130	-1.99373
2012	11.38349	4.080285	14.59365	-1.806800	2.397895	1.524667	9.515469	0.591130	-1.64018
2013	11.37969	4.080285	14.5241	-1.806800	2.397895	1.524667	10.16812	0.591130	-0.95973
2014	11.40961	4.080285	14.51607	-1.806800	2.60269	1.524667	10.36845	0.591130	-0.99188
2015	11.4666	4.080285	14.55458	-1.806800	2.778819	1.524667	10.39821	0.591130	-1.11971
2016	4.708798	4.080285	14.52467	-1.806800	2.639057	1.524667	10.44874	0.591130	-1.33983
2017	4.831022	4.080285	14.51633	-1.806800	2.302585	1.524667	10.50342	0.591130	-1.21103
2018	4.938758	4.080285	14.50792	-1.806800	2.302585	1.524667	10.6079	0.591130	-1.41307
2019	5.013293	4.080285	14.49945	-1.806800	1.99243	1.524667	10.67089	0.591130	-1.53688

**Table 4:** The VECM model.

Year	LM2	b	LY	c	LR	d	LEXR	e	Lyu
1997	10.15759	-10.09675	13.95394	2.668756	2.079442	5.124554	8.39976	-4.763585	-2.09209
1998	10.1404	-10.09675	13.97412	2.668756	2.079442	5.124554	8.472614	-4.763585	-1.7148
1999	10.15157	-10.09675	13.99319	2.668756	2.079442	5.124554	8.774622	-4.763585	-1.29316
2000	10.1518	-10.09675	14.0244	2.668756	2.079442	5.124554	9.063463	-4.763585	-1.8452
2001	10.28995	-10.09675	14.06445	2.668756	2.079442	5.124554	9.003439	-4.763585	-1.24927
2002	10.43549	-10.09675	14.08435	2.668756	1.94591	5.124554	8.977778	-4.763585	-1.29913
2003	10.55196	-10.09675	14.16128	2.668756	1.94591	5.124554	8.986071	-4.763585	-2.09049
2004	10.63859	-10.09675	14.24199	2.668756	1.94591	5.124554	9.026778	-4.763585	-2.08849
2005	10.76126	-10.09675	14.27501	2.668756	1.94591	5.124554	9.076466	-4.763585	-2.09049
2006	10.95765	-10.09675	14.31977	2.668756	1.94591	5.124554	9.109636	-4.763585	-2.09049
2007	11.17778	-10.09675	14.374	2.668756	1.94591	5.124554	9.129781	-4.763585	-2.09049
2008	11.25332	-10.09675	14.46114	2.668756	1.94591	5.124554	9.14388	-4.763585	-2.07422
2009	11.17492	-10.09675	14.47085	2.668756	2.197225	5.124554	9.176473	-4.763585	-2.06638
2010	11.28712	-10.09675	14.49321	2.668756	2.197225	5.124554	9.208238	-4.763585	-1.72252
2011	11.39475	-10.09675	14.55606	2.668756	1.791759	5.124554	9.268704	-4.763585	-1.99373
2012	11.38349	-10.09675	14.59365	2.668756	2.397895	5.124554	9.515469	-4.763585	-1.64018
2013	11.37969	-10.09675	14.5241	2.668756	2.397895	5.124554	10.16812	-4.763585	-0.95973
2014	11.40961	-10.09675	14.51607	2.668756	2.60269	5.124554	10.36845	-4.763585	-0.99188
2015	11.4666	-10.09675	14.55458	2.668756	2.778819	5.124554	10.39821	-4.763585	-1.11971
2016	4.708798	-10.09675	14.52467	2.668756	2.639057	5.124554	10.44874	-4.763585	-1.33983
2017	4.831022	-10.09675	14.51633	2.668756	2.302585	5.124554	10.50342	-4.763585	-1.21103
2018	4.938758	-10.09675	14.50792	2.668756	2.302585	5.124554	10.6079	-4.763585	-1.41307
2019	5.013293	-10.09675	14.49945	2.668756	1.99243	5.124554	10.67089	-4.763585	-1.53688

LM2: Liquidity logarithm, b: Coefficient, LY: Logarithm of GDP, c: Coefficient, LR: Interest rate, d: Coefficient, LEXR: Logarithm of the exchange rate, e: Coefficient, LYu: Logarithm of the underground economy.

MATLAB was used for the computations. Membership functions were defined as triangular and trapezoidal. Afterwards, the program was run. Then debugging was performed and eventually, the variables were defuzzified, and fuzzy was completed. The size of the shadow economy was calculated based on the percentage of GDP [9].

(TR): Tax burden rate

The difference between this article and the article by Hui Kuang Yu et al. (2006) is that here, the tax burden rate and the exchange rate margin were used as input variables. But, in the article by Hui Kuang Yu et al., the variables of effective tax rate and the degree of government regulation were used. The novelty of this paper is that all the input variables and fuzzy rules were given to the MATLAB software and the software performed the estimation. Then it examined the effect of the underground economy on the money demand. The results are presented in the following tables.

## Conclusion

First, the size of the underground economy was estimated. For this purpose, the fuzzy method was used. The underground economy had a negative and insignificant impact in the short term. But, its effect in the long run was positive and significant. This indicates that the underground economy has a direct effect on the amount of dirty money.

## Declaration of Competing Interest

The authors declare that they have no known competing for financial interests or personal relationships that could have appeared to influence the work reported in this article.

## Appendix Supplementary materials

Image, application 1
